# Exploring the Model of Cefazolin Released from Jellyfish Gelatin-Based Hydrogels as Affected by Glutaraldehyde

**DOI:** 10.3390/gels10040271

**Published:** 2024-04-17

**Authors:** Wiriya Charoenchokpanich, Pratchaya Muangrod, Sittiruk Roytrakul, Vilai Rungsardthong, Benjamaporn Wonganu, Sawanya Charoenlappanit, Federico Casanova, Benjawan Thumthanaruk

**Affiliations:** 1 Department of Agro-Industrial, Food, and Environmental Technology, Faculty of Applied Science, King Mongkut’s University of Technology North Bangkok, Bangkok 10800, Thailand; w.charoenchokpanich@outlook.com (W.C.); p.muangrod@outlook.com (P.M.); vilai.r@sci.kmutnb.ac.th (V.R.); 2 Functional Proteomics Technology Laboratory, National Science and Technology Development Agency (NSTDA), Pathum Thani 12120, Thailand; sittiruk@biotec.or.th (S.R.); sawanya.cha@ncr.nstda.or.th (S.C.); 3 Food and Agro-Industry Research Center, King Mongkut’s University of Technology North Bangkok, Bangkok 10800, Thailand; 4 Center for Food Industry Innovation Technology, King Mongkut’s University of Technology North Bangkok, Bangkok 10800, Thailand; 5 Department of Biotechnology, Faculty of Applied Science, King Mongkut’s University of Technology North Bangkok, Bangkok 10800, Thailand; benjamaporn.w@sci.kmutnb.ac.th; 6 Research Group for Food Production Engineering, National Food Institute, Technical University of Denmark, 28000 Kongens Lyngby, Denmark; feca@food.dtu.dk

**Keywords:** jellyfish, gelatin-based hydrogels, glutaraldehyde, drug delivery, cefazolin

## Abstract

Due to its excellent biocompatibility and ease of biodegradation, jellyfish gelatin has gained attention as a hydrogel. However, hydrogel produced from jellyfish gelatin has not yet been sufficiently characterized. Therefore, this research aims to produce a jellyfish gelatin-based hydrogel. The gelatin produced from desalted jellyfish by-products varied with the part of the specimen and extraction time. Hydrogels with gelatin: glutaraldehyde ratios of 10:0.25, 10:0.50, and 10:1.00 (*v*/*v*) were characterized, and their cefazolin release ability was determined. The optimal conditions for gelatin extraction and chosen for the development of jellyfish hydrogels (JGel) included the use of the umbrella part of desalted jellyfish by-products extracted for 24 h (WU24), which yielded the highest gel strength (460.02 g), viscosity (24.45 cP), gelling temperature (12.70 °C), and melting temperature (22.48 °C). The quantities of collagen alpha−1(XXVIII) chain A, collagen alpha−1(XXI) chain, and collagen alpha−2(IX) chain in WU24 may influence its gel properties. Increasing the glutaraldehyde content in JGel increased the gel fraction by decreasing the space between the protein chains and gel swelling, as glutaraldehyde binds with lateral amino acid residues and produces a stronger network. At 8 h, more than 80% of the cefazolin in JGel (10:0.25) was released, which was higher than that released from bovine hydrogel (52.81%) and fish hydrogel (54.04%). This research is the first report focused on the production of JGel using glutaraldehyde as a cross-linking agent.

## 1. Introduction

One of the most common problems after an operation is microbial infections, which can endanger patients’ lives, prolong the wound healing process, and may even be lethal [1,2]. The most common microbial infection-causing bacteria are *Staphylococcus aureus* and *Streptococcus pyogenes*. In general, antibiotics are required for the treatment of wound infections. Cefazolin is recommended and widely used as an antimicrobial prophylactic drug after surgical operations. Cefazolin is effective against Gram-positive and Gram-negative organisms such as *Staphylococcus aureus* and *Escherichia coli*, and it acts by inactivating penicillin-binding proteins on the inner membrane of the bacterial cell wall [3]. However, the global problem of antibiotic resistance due to the overuse of antibiotics has become a severe concern [4]. If there is wound infection, optimizing wound healing by preventing bacterial colonization with a dressing incorporated with an antibacterial drug could be a method of choice [5]. As a result, wound dressings should include antifouling and antibacterial properties to protect against wound site infections. In this context, hydrogels are some of the most effective options for antibacterial wound dressings because of their intrinsic drug-delivery capacity [6].

Hydrogels are three-dimensional (3D) polymeric units that absorb large amounts of water and other biological fluids, maintaining moisture around the wound site [7]. Hydrogels have gained attention for their potential use in biomedical fields, including tissue engineering and biomedicine [8]. However, the application of hydrogels in biomedical fields could still be improved, considering their excellent biocompatibility and ease of biodegradation [9,10]. Hydrogels based on biopolymers, such as cellulose [11,12] and chitosan [13], and proteins, especially gelatin [7,14], have been developed. Glutaraldehyde has been extensively used as a cross-linking agent in gelatin-based hydrogels, as it easily reacts with gelatin; in particular, it promotes binding with the lateral amino acid residues or N-terminal amino acid of gelatin [15], resulting in a stronger network. Gelatin-based hydrogels may decrease drug resistance through direct action on the specific site and improve the bioavailability of medication through reducing the dosing frequency, thus promoting patient compliance and convenience [16].

Gelatin is a biopolymer produced through the thermal denaturation of collagen [17] and is the only protein-based hydrocolloid. The main difference between gelatin and other hydrocolloids is its cold temperature gel-setting and thermo-reversible properties. Additionally, gelatin has prominent advantages over other gelling agents and has been extensively utilized in the food, biomedicine, and cosmetic industries [18]. Gelatin is commonly used in biomedicine due to its excellent biocompatibility and biodegradability [7]. The utilization of marine gelatin has gained increased interest, as the properties of marine gelatin are comparable to those of mammalian gelatins (e.g., bovine gelatin and porcine gelatin) to some extent. Marine gelatins are mainly produced from by-products, considering their functional properties and decreased food waste.

In Thailand, white-type jellyfish (*Lobonema smithii*) are produced and exported in a dried and salted form to several countries, including China, Japan, South Korea, and Taiwan [19]. Many salted jellyfish by-products are formed in processing, such as broken pieces or irregular shapes, which have gained increasing attention in gelatin extraction research. The salted jellyfish by-product has a low price but is still rich in protein (3.61%), especially collagen [20]. Apart from the collagen in jellyfish, medical benefits—including those related to high blood pressure, arthritis, and fatigue—have been associated with jellyfish samples [21,22,23,24]. Several studies on the production of jellyfish gelatin have been reported. The importance of gelatin depends on the gel properties, including gelling properties (gelling and melting temperature), viscosity, and gel strength. Factors affected by production yields and gelatin quality depend on the studied marine sources, pre-treatment, and extraction temperature and time [25]. The effects of extraction methods on the physical properties of jellyfish gelatin have been systematically studied to a limited extent. Previous studies have reported that the strength of the resulting jellyfish gelatin gel was lower than that of other commercial gelatins produced from bovine, porcine, and marine sources. Cho et al. [26] reported that the gel strength of jellyfish (*Rhopilema hispidum*) gelatin was 31.2 kPa, while Lueyot et al. [27] reported that jellyfish by-product (*Lobonema smithii*) gelatin extracted at 60 °C for 12 h showed a maximum gel strength of 323.74 g. However, the cold setting and thermo-reversible gel properties of such gelatin could benefit medical gel applications. To date, no jellyfish gelatin has been utilized as a hydrogel for use in a drug release system. Therefore, the objectives of this research were to characterize the quality of a jellyfish gelatin-based hydrogel developed using glutaraldehyde, as well as to compare the effect of glutaraldehyde on the release of cefazolin from bovine, fish, and jellyfish hydrogels.

## 2. Results and Discussion

### 2.1. Quality of Jellyfish By-Product Gelatin Extracted from Different Parts of Jellyfish and Extraction Times

#### 2.1.1. Gelatin Yield

The extraction yields from the umbrella part of the desalted jellyfish by-product at 60 °C for 24 (WU24) and 48 (WU48) h were 19.77 ± 0.88% and 29.73 ± 0.63%, respectively. The extraction yields from the oral arm part of the desalted jellyfish by-product at 60 °C for 24 (WO24) and 48 (WO48) h were 28.29 ± 0.33% and 39.04 ± 0.03%, respectively. Therefore, the gelatin yield extracted from the umbrella part was higher than that extracted from the oral arm of the jellyfish, and the gelatin yields significantly increased with an increased extraction time. The gelatin yield of the WO48 sample was higher than that of the other sample. Compared to the same desalted jellyfish by-product, the sample was washed using a sonication bath, pre-treated with 0.2 M hydrochloric acid (HCl), and extracted at 80 °C for 4, 6, and 8 h. The results indicated that the gelatin yield was 7.43–32.69%. The highest gelatin yield obtained in this study was similar to that for the sample extracted at 80 °C for 8 h, at 32.69% [28]. The gelatin yield of the WO48 sample was higher than that in the previous work, which may have resulted from the raw material and extraction time. The increased extraction temperature and time in the gelatin extraction process could destabilize the covalent bonds in the collagen network, allowing free α- and β-chains to be liberated from the protein [29].

#### 2.1.2. Gel Strength 

The gel strength of 6.67% gelatin (commercial bovine gelatin, BG; fish gelatin, FG; and jellyfish by-product gelatin) is presented in Table 1. The gel strength of gelatin extracted from the desalted jellyfish by-product (umbrella and oral arm parts) at 60 °C for 24 and 48 h ranged from 408.08 to 460.02 g. The highest gel strength was obtained for the WU24 sample (460.02 g), while the lowest was the WO48 sample (408.08 g). The gelatin extracted from the umbrella part of the desalted jellyfish by-product had a higher gel strength than that from the oral arm part. Moreover, the gel strength of jellyfish by-product gelatin extracted at 60 °C for 24 h (449.11–460.02 g) was higher than that extracted at 60 °C for 48 h (408.08–412.63 g), with the gel strength decreasing as the extraction time increased. However, the gel strength values of all jellyfish by-product gelatin gel samples considered in this research were lower than those of commercial bovine (846.31 g) and fish (625.55 g) gelatin. Lueyot et al. [27] reported that the gel strength of jellyfish gelatin was lower than bovine and fish gelatin, which may be due to differences in the collagen type, amino acid composition, α-chain to β-chain ratio, and α-chain peptides. Furthermore, imino acids (hydroxyproline and proline) influence the formation of gels. The imino acid content in mammalian gelatin was reportedly higher than that in marine gelatin [30]. Compared to gelatin extracted from jellyfish in previous works, the gel strength of the WU24 sample in this work was higher than that of the desalted jellyfish by-product gelatin after the sample was treated with 0.1 M HCl and extracted at 60 °C for 12 h (323.74 g) [27]; the desalted jellyfish by-product gelatin obtained when the sample was washed using a sonication bath for 40 min, pre-treated with 0.2 M HCl, and extracted at 80 °C for 4 h (447.01 g) [28]; and cannonball jellyfish gelatin soaked in 1.5% citric acid and extracted at 60 °C for 4.5 h (3.4 g) [31]. Compared to other marine gelatin samples, the gel strength of the WU24 sample showed lower strength than that of the tilapia skin gelatin after the sample was pre-treated with pepsin enzyme (5 units/g of the sample) and extracted at 55 °C for 6 h (725 g) [32]. Therefore, the factors affecting the gel strength of extracted gelatin include different raw materials, pre-treatment, extraction temperature, and extraction times, which influence the conversion of collagen into gelatin and affect the gel’s quality. The molecular weight (MW) distribution and concentration, the size of the protein chain, and the amino acid composition of the obtained gelatin also influence its qualities.

#### 2.1.3. Viscosity 

The viscosity values for the commercial gelatins and the jellyfish by-product gelatin are shown in Table 1. The viscosity of the jellyfish by-product gelatin ranged from 20.61 to 24.45 cP. The highest viscosity value was obtained for the WU24 sample (24.45 cP), while the lowest was for the WO48 sample (20.61 cP). The gelatin extracted from the umbrella part of desalted jellyfish by-products (22.44–24.45 cP) had a higher viscosity than that from the oral arm parts (20.61–23.62 cP), and the viscosity value decreased as the extraction time increased. The viscosity of jellyfish by-product gelatins was also lower than that of commercial bovine (30.06 cP) and fish (25.54 cP) gelatin. Compared to other gelatins, the viscosity of all jellyfish by-product gelatin samples ranged from 2.0 to 13.0 cP [18,33], while the viscosity of commercial bovine and fish gelatin was 30.06 cP and 25.54 cP, respectively. These results differed from other studies, which reported the viscosity of commercial bovine gelatin as 9.8 cP [18]. However, the viscosity of the jellyfish by-product gelatin in this work was higher than previously reported. Specifically, the highest viscosity of jellyfish by-product gelatin was 7.73 cP when extracted at 60 °C for 12 h [27]. The viscosity of the jellyfish by-product gelatin was consistent with the protein profile, in which the longer extraction time resulted in increased short-chain protein or low-MW peptides and low gelling ability.

#### 2.1.4. Gelling Properties

The gelling temperature and melting temperature of gelatin are other essential characteristics of gelatin that affect its storage stability. Temperature sweep tests can be used to determine the gelling and melting temperatures of gelatin gel, in which the high temperature (40 °C) is changed to a low temperature (5 °C), and vice versa, using a stress-controlled rheometer [18]. The dynamic viscoelastic profile changes in gelatins during gelling and melting are presented in Figure S1 (Supplementary Materials Figure S1). The gelatin gels are formed when gelatin chains are changed from single-strand to triple-helix due to ionic interactions, hydrophobic association, hydrogen bonding, and Van der Waals forces [34]. Table 1 shows that the gelling and melting temperatures of the jellyfish by-product gelatin were 8.56–12.70 °C and 18.18–22.48 °C, respectively. The gelling and melting temperatures of the jellyfish by-product gelatin were consistent with the measured gel strength and viscosity, where the thermal stability of the gelatin gel decreased as the extraction time increased. The highest gelling and melting temperatures of jellyfish by-product gelatin were obtained for the WU24 sample. Compared to commercial bovine and fish gelatin, the gelling temperature of commercial fish gelatin was lower than that of both commercial bovine gelatin (22.22 °C) and commercial fish gelatin (18.69 °C); furthermore, the melting temperature of jellyfish by-product gelatin was lower than that of both commercial bovine gelatin (30.23 °C) and commercial fish gelatin (25.23 °C). The gelling properties of jellyfish by-product gelatin were lower than those of commercial gelatin, which may be due to the jellyfish by-product gelatin samples exhibiting a low MW, short-chain peptides, and a low α-chain content [35], resulting in low functionalities compared to commercial gelatin. Despite its low thermal properties, jellyfish by-product gelatin could be utilized in refrigerated and frozen food products, as well as pharmaceutical products such as hydrogels. 

#### 2.1.5. Fourier Transform Infrared (FTIR) Spectra

**Figure 1:**
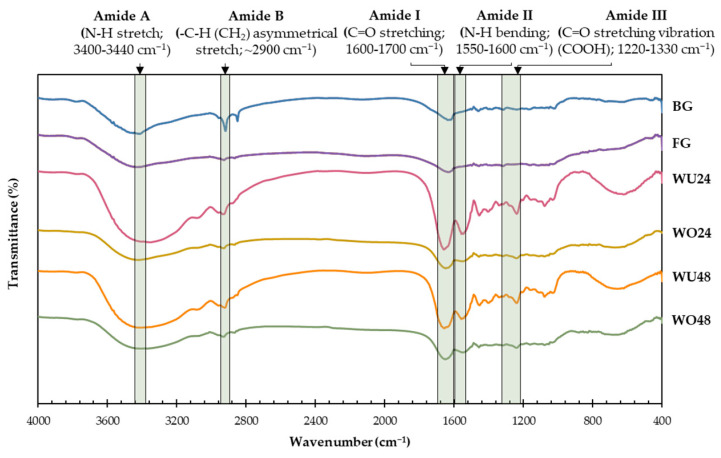
FTIR spectra of commercial bovine gelatin, commercial fish gelatin, and jellyfish by-product gelatin obtained with different parts of jellyfish and extraction times.

FTIR spectroscopy was used to investigate changes in the secondary protein structure at the amide region of gelatin. The amide A, B, I, II, and III patterns in commercial gelatin and all jellyfish by-product gelatins were monitored, as shown in Figure 1, whereas the FTIR peak locations are presented in Table S1 (Supplementary Materials Table S1). The FTIR spectra patterns for jellyfish by-product gelatin changed with the duration of the gelatin extraction time. Amide A occurs in the 3400–3440 cm^−1^ range and is related to a N-H stretching vibration, while amide B is related to the asymmetric stretching vibration of =C-H and NH_3_^+^. The position shifts of amide A and B bands to a lower wavenumber indicate that the N-H group of peptides participates in hydrogen bonds, and high-temperature extraction enhances the interactions of -NH_3_^+^ groups between peptide chains [28]. Thus, the high-temperature extraction destroyed hydrogen bonds and broke collagen structures during gelatin extraction [36].

The amide I vibrations were analyzed to predict the secondary protein structure. The amide I bands were observed in a spectrum between 1600 and 1700 cm^−1^, associated with the C=O stretching vibration. The amide I bands of WU24, WO24, WU48, and WO48 were observed at 1626.89, 1628.54, 1630.84, and 1630.72 cm^−1^, respectively. The wavenumber of amide I for jellyfish by-product gelatin was higher than that of commercial bovine (1607.43 cm^−1^) and fish (1619.56 cm^−1^) gelatin. The amide I band shifted to a higher wavenumber when the extraction temperature and time increased. The increased extraction time could cause a loss of the triple-helix of the protein collagen, resulting in increased disintegration of the alpha-helix structure into a random coil (Table S2; Supplementary Materials Table S2). Muyonga et al. [37] reported that gelatin extraction with an increased temperature and time caused the molecules to shift from an alpha-helical structure to a random coil, denaturing the collagen protein into gelatin. The amide II wavenumbers of the WU24, WO24, WU48, and WO48 samples were observed at 1558.84, 1559.08, 1555.08, and 1550.02 cm^−1^, respectively. Amide II is associated with N-H bending vibration and C-N stretching vibrations. Amide II bands of jellyfish by-product gelatin were higher than that of commercial bovine (1561.36 cm^−1^) and fish (1567.14 cm^−1^) gelatin. The lower wavenumber of jellyfish by-product gelatin indicated more intense NH bonding in hydrogen [38]. Amide III is associated with the structure of triple helical collagen, which is located at 1220–1330 cm^−1^ [37]. The amide III wavenumbers of WU24, WO24, WU48, and WO48 were 1239.29, 1238.63, 1229.95, and 1229.07 cm^−1^, respectively. The amide III bands of jellyfish by-product gelatin samples had a lower wavenumber as the gelatin extraction temperature and time increased. Lueyot et al. [28] reported that the amide I and III band intensities decreased with gelatin extraction time, and the extraction time was associated with the loss of molecular order in jellyfish gelatin. These results indicated that the higher degradation of jellyfish by-product gelatin extracted at higher temperatures and times affected the protein structure of the jellyfish by-product, including the α-chain, triple-helix structure, and functional groups in jellyfish by-product gelatin. Therefore, the increased extraction times resulted in increased gelatin yield but lower gelling properties.

#### 2.1.6. Protein Pattern 

**Figure 2:**
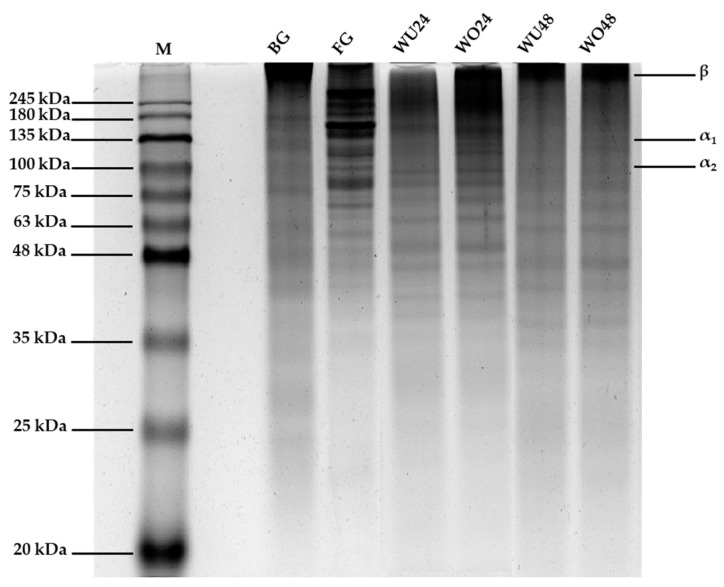
Protein pattern of molecular weight standard (M) and jellyfish by-product gelatin from different parts of jellyfish and extraction times compared to commercial gelatins (bovine, BG; and fish, FG).

The protein pattern of gelatin (1000 µg) extracted from the umbrella and oral arm part of desalted jellyfish by-product extracted at 60 °C for 24 and 48 h is compared with that of commercial bovine and fish gelatin in Figure 2. The protein patterns of commercial bovine and fish gelatin samples revealed a protein MW ranging from 25–245 kDa to 48–245 kDa, respectively. Meanwhile, a MW of 40–180 kDa was observed in WU24 and WO24, whereas a MW of 35–180 kDa was found in WU48 and WO48. Moreover, α_1_- and α_2_-chains were observed in all samples, with MWs of 135 and 113 kDa, respectively. This protein pattern is similar to that of the SDS-PAGE gel of Klaiwong et al. [39], where the α_1_ and α_2_ bands exhibited collagen protein at 135 kDa and 113 kDa, respectively. The protein patterns of gelatin extracted from the umbrella and oral arm parts of the white-desalted jellyfish by-product did not differ. However, gelatin extracted from white-desalted jellyfish by-product at 60 °C for 24 h was found to have a MW higher than that of gelatin extracted at 60 °C for 48 h. Thus, the MW decreased with increasing extraction time. As the extraction time increased, the major protein constituents (α-, β-, and γ-chain) of the gelatin degraded, increasing short-chain proteins or low-MW peptides. In addition, the interchain cross-links of collagen were hydrolyzed during gelatin extraction. Consequently, gelatin has peptides with varying MWs [40]. The MW distribution, structure, and subunit composition of gelatins—including α-chain and low-MW protein fragments (~35 kDa)—affect its physical and functional properties [30]. 

#### 2.1.7. Identification of Proteins in Gelatin

**Figure 3:**
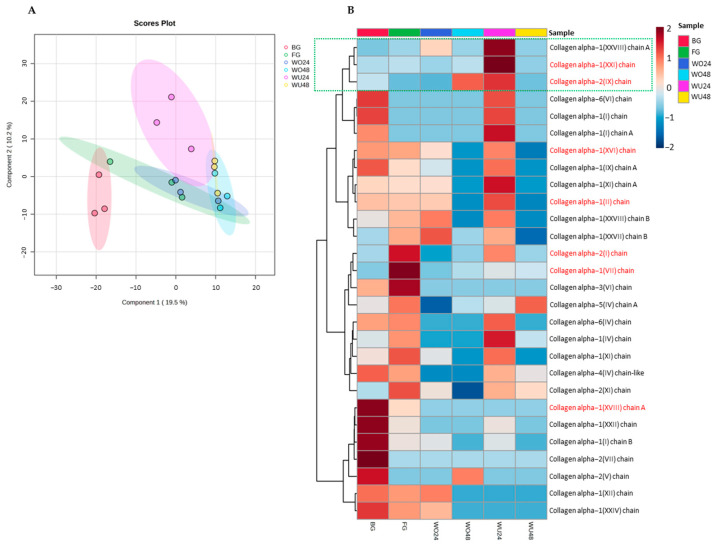
The relative amount of each protein found in the gelatin samples. (**A**) Partial least squares-discriminant analysis (PLS-DA) of protein in gelatin samples and (**B**) heat maps of protein types in each gelatin sample.

The peptides in gelatin were identified using LC-MS/MS. Different amino acid sequences and collagen types may explain the gel properties of commercial gelatin and jellyfish by-product gelatin obtained with different raw materials and extraction times. Protein names and the sequence of commercial gelatins (bovine, BG; and fish, FG) and jellyfish by-product gelatin with different parts of jellyfish and extraction times are presented in Table S3 (Supplementary Materials Table S3). The relative amount of each protein found in the gelatin samples is shown in Figure 3. A total of 28 peptides was identified in commercial (bovine and fish) gelatin and jellyfish by-product gelatin samples. However, the protein types in BG, FG, WU24, WO24, WU48, and WO48 samples differed in terms of peptides and quantities in each sample (Figure 3A), due to the differences between samples and extraction times. The differences in peptides between each sample affected the gel strength, viscosity, melting temperature, and gelling temperature of the gelatin. The gelatin peptide chain was shorter when the extraction time increased. The identification and quantity of proteins in gelatin were consistent with the gel properties under longer extraction times, resulting in increased short-chain proteins and changes in the collagen polypeptide chain [41].

Proteomic analysis showed that the peptide identification in jellyfish by-product gelatin samples was lower than in commercial gelatin. In the BG, FG, WU24, WO24, WU48, and WO48 samples, 28, 23, 22, 16, 11, and 10 peptides were identified, respectively (BG > FG > WU24 > WO24 > WU48 > WO48). At least seven peptide chains—identified as collagen alpha-1(II) chain, collagen alpha-1(VII) chain, collagen alpha-1(XVI), collagen (XXI) chain, collagen alpha-1(XXVIII) chain A, collagen alpha-2(I) chain, and collagen alpha-2(IX) chain—were similarly found in commercial gelatins and jellyfish by-product gelatin, one of which contained collagen alpha-2(I) chain. The sequence of the collagen alpha-2(I) chain was GDPGPPGL. Comparing the jellyfish by-product gelatin, the collagen alpha-2(I) chain quantity in WU24 was higher than that in WO24, WU48, and WO48. However, when compared with commercial gelatins, the collagen alpha-2(I) chain quantity in WU24 was lower than that in the FG sample (Figure 3B). Lueyot et al. [27] reported that the collagen alpha-2(I) and collagen alpha-2(IV) chains are unique peptide sequences that influence the gel properties that are found in bovine and fish gelatin. The differences between protein types and quantities of protein in the commercial gelatins and jellyfish by-product gelatin revealed that WU24 was more closely related to the commercial gelatins than the other jellyfish by-product gelatins. However, collagen alpha-1(XVIII) chain A, collagen alpha-1(XXIII) chain, collagen alpha-1(I) chain B, collagen alpha-2(VII) chain, collagen alpha-2(V) chain, collagen alpha-1(XIII) chain, and collagen alpha-1(XXIV) chain in the jellyfish by-product gelatin WU24 had lower relative abundance values than in the commercial gelatins, especially bovine gelatin, thus exhibiting lower gel qualities. Interestingly, the quantities of collagen alpha-1(XXVIII) chain A (GPAGPSGMQGFP), collagen alpha-1(XXI) chain (PGDSGEPGDKG), and collagen alpha-2(IX) chain (VGKPGGKG) in WU24 were higher than in the commercial gelatins and the other jellyfish by-product gelatins, which may result in the gel qualities of WU24 being higher than that of the other jellyfish by-product samples. Therefore, the inferior functional gel properties of jellyfish by-product gelatin may be due to differences in the type and quantity of collagen. 

### 2.2. Quality of Hydrogels

#### 2.2.1. Gel Fraction and Porosity 

Determining the gel fraction allows for qualitative analysis of the network formation efficiency in hydrogels. The gel fraction indicates the number of cross-links between the hydrogel polymers and the amount of insoluble gel in water. The gel fraction percentages of the gelatin-based hydrogels are shown in Table 2. The gel fraction percentages of the JGel0.25, JGel0.50, and JGel1.00 samples were 12.58%–17.20%. For commercial gelatin, the gel fractions of bovine hydrogels (BGel0.25, BGel0.50, and BGel1.00) and fish hydrogels (FGel0.25, FGel0.50, and FGel1.00) were 14.97%–19.14% and 13.02%–17.32%, respectively. The gel fraction percentage increased with an increasing concentration of glutaraldehyde. Compared to commercial bovine and fish gelatin-based hydrogels, the gel fraction of JGel was lower. Formulating gelatin-based hydrogels with glutaraldehyde is chemical cross-linking. The mechanism of gelatin cross-linking using glutaraldehyde involves the nucleophilic addition-type reaction between the aldehyde functional groups and free non-protonated ε-amino groups (-NH_2_) of the lysine or hydroxylysine of gelatin. The reaction starts with the nucleophilic addition of the ε-NH_2_ groups to the aldehyde’s carbonyl groups (C=O) to form a tetrahedral unstable carbinolamine intermediate. Then, the protonation of the -OH group and loss of a water molecule yields the conjugated Schiff’s bases [42] (Figure S2; Supplementary Materials Figure S2). Therefore, the increased bonding between glutaraldehyde and gelatin with the lateral amino acid residues—especially with the ε-amino group of lysine and hydroxylysine or N-terminal amino acid in gelatin Schiff’s base reaction [15]—results in high cross-linking and a high gel fraction [43].

The hydrogel porosity dictates a drug’s effectiveness in terms of absorbing into or releasing from the hydrogel. The gel porosities of the gelatin-based hydrogels are shown in Table 2. The gel porosity of the JGel0.25, JGel0.50, and JGel1.00 samples was 1.83%–2.20%, with the volume of porosity for JGel0.25, JGel0.50, and JGel1.00 being 92.40 mm^3^, 80.22 mm^3^, and 76.86 mm^3^, respectively. The gel porosity values of jellyfish hydrogels were higher than that of the commercial gelatin-based hydrogels. The gel porosity of bovine hydrogels (BGel0.25, BGel0.50, and BGel1.00) and fish hydrogels (FGel0.25, FGel0.50, and FGel1.00) were 1.73%–1.92% and 1.79%–2.15%, respectively. The volume of porosity for BGel0.25, BGel0.50, and BGel1.00 was 80.64 mm^3^, 78.96 mm^3^, and 72.66 mm^3^, respectively; while the volume of porosity in FGel0.25, FGel0.50, and FGel1.00 was 90.30 mm^3^, 79.80 mm^3^, and 75.18 mm^3^, respectively. The increasing content of glutaraldehyde in hydrogels results in a higher gel formation percentage, a closer network of polymer chains, and an altered hydrogel network structure that makes the network more robust [44]. Therefore, the gel porosity decreased as the ratio of glutaraldehyde in hydrogel increased.

#### 2.2.2. Microstructure

**Figure 4:**
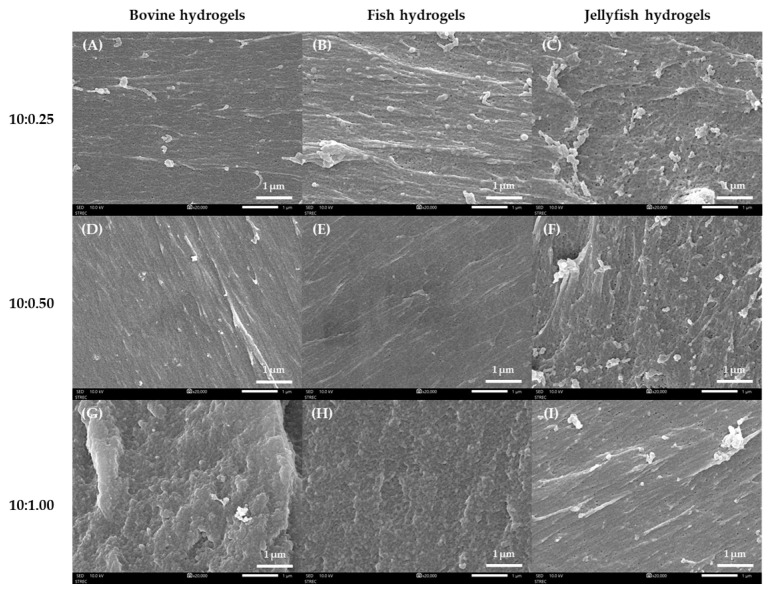
The cross-section of hydrogel crosslinked with glutaraldehyde was magnified 20,000 times. (**A**) BGel0.25, (**B**) FGel0.25, (**C**) JGel0.25, (**D**) BGel0.50, (**E**) FGel0.50, (**F**) JGel0.50, (**G**) BGel1.00, (**H**) FGel1.00, and (**I**) JGel1.00.

The microstructure of gelatin-based hydrogels with glutaraldehyde as a cross-linking agent was observed using SEM, as shown in Figure 4. The micrographs of the gelatin-based hydrogels showed dispersed pores on the surface of hydrogels, which differed depending on the glutaraldehyde content. The pore size and space between the protein chains of jellyfish hydrogels, bovine hydrogels, and fish hydrogels showed a similar pattern. The hydrogel’s pore size was influenced by the glutaraldehyde content, as increased glutaraldehyde content in a hydrogel decreases the distance between protein chains, resulting in a more solid-like structure and a strong network [44,45]. 

The hydrogel’s pore size decreased from loosely cross-linked hydrogels in Gel0.25 (Figure 4A–C) to densely cross-linked hydrogels in Gel0.50 (Figure 4D–F), and there was no apparent porous structure in Gel1.00 (Figure 4G–I). These results were consistent with the gel porosity values and indicated that the pore size of hydrogel decreased with increased glutaraldehyde content in hydrogels. Munaini et al. [45] reported that the microstructure of porcine hydrogels with the lowest glutaraldehyde content (25 µL) showed an internal structure close to that of untreated gelatin, which has regular and organized porosities. Increased glutaraldehyde content resulted in the loss of pores and decreased space between the gelatin chains due to a more inhomogeneous structure.

#### 2.2.3. Gel Swelling

**Figure 5:**
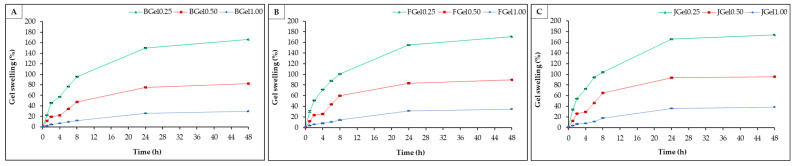
Gel swelling of the gelatin-based hydrogel. (**A**) Bovine hydrogels, (**B**) fish hydrogels, and (**C**) jellyfish hydrogels.

The swelling of gelatin-based hydrogels soaked with phosphate buffer (PBS) with a pH of 7.4 is shown in Figure 5. The gel swelling increased as soaking time increased, and reached an equilibrium value after 24 h. In the first 24 h, the swelling of jellyfish hydrogels (36.00%–165.74%), bovine hydrogels (25.91%–150.03%), and fish hydrogels (31.13%–155.00%) increased rapidly. The swelling of hydrogels was caused by the hydrogen (H^+^) in the polymer chains reacting with water to create a positive charge (H_3_O^+^), and the polymer chains became more negatively charged. The increased negative charge density triggers electrostatic repulsion, causing the polymer chains to stretch. Then, H^+^ in water—which has a positive charge—reacts with the negative charge in polymer chains, forming hydrogen bonds in the polymer chain [46].

Consequently, more pores or space between protein chains allow for more absorption of water molecules, exhibiting gel swelling. The swelling of jellyfish hydrogels, bovine hydrogels, and fish hydrogels showed a similar trend. However, the swelling of jellyfish hydrogel was higher than that of bovine hydrogels and fish hydrogels, due to the higher MW of commercial gelatin, resulting in strong intermolecular interactions and a closer network polymer chain [44,47]. Therefore, the commercial gelatin-based hydrogel can absorb a lower solution. Compared to the ratio of glutaraldehyde, the gel swelling of the hydrogel sample formed when 10% gelatin was cross-linked with 5% glutaraldehyde at 10:0.25 (i.e., Gel0.25) presented the highest swelling. The gel swelling of the hydrogel decreased as the content of glutaraldehyde increased. Increasing the glutaraldehyde content in hydrogel reduced the space between polymer protein chains and produced a dense network. These results were consistent with the gel porosity and microstructure results, which decreased with an increase in the ratio of glutaraldehyde.

#### 2.2.4. In Vitro Drug Release 

**Figure 6:**
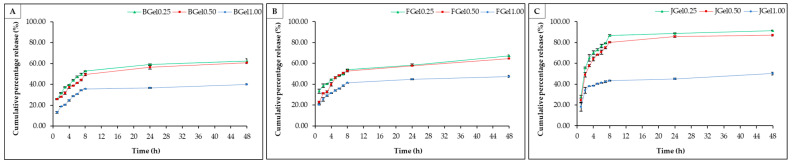
Cumulative percentage release of cefazolin in hydrogels. (**A**) Bovine hydrogels, (**B**) fish hydrogels, and (**C**) jellyfish hydrogels.

A cefazolin solution was loaded into the hydrogels using the in situ method, which incorporated 100% of the drug into the hydrogel. Loading the cefazolin solution into hydrogels during the gel formation process resulted in the gelatin-based hydrogel absorbing the cefazolin solution, which reacts with hydrophilic groups such as -NH_2_, -COOH, and -OH) at the covalent or hydrogen bonds [48] (Figure S3; Supplementary Materials Figure S3). The drug release profiles of jellyfish hydrogels, bovine hydrogels, and fish hydrogels cross-linked with glutaraldehyde are shown in Figure 6. Drug release occurs through the diffusion from high to low concentration [45]. Polymer corrosion results in drug release from the hydrogel, possibly due to the cauterized gel fraction. In the first 8 h, cefazolin in the hydrogels was released rapidly, while its slow release from the hydrogel was also observed due to the stable distribution of the cefazolin in the hydrogels. The cefazolin release rate from jellyfish hydrogel and commercial (bovine and fish) gelatin-based hydrogels showed a similar trend. The cumulative percentage release (CPR) of cefazolin from jellyfish hydrogels (43.44%–86.77%) was higher than that for bovine hydrogels (36.65%–54.05%) and fish hydrogels (41.30%–52.82%), when soaked with PBS with pH of 7.4 at 37 °C for 8 h. The increase in glutaraldehyde added into the gelatin gel affected the hydrogel swelling, thereby delaying the release of cefazolin. The cefazolin in the Gel1.00 sample showed a lower release than that from the Gel5.00 and Gel0.25 samples. The release pattern of the drug cefazolin seemed similar in Gel0.25 and Gel0.50, which might be due to the direct loading of cefazolin directly into the hydrogels, resulting in the structure of the hydrogel (Gel0.25 and Gel0.50) exhibiting instability and the surface of hydrogel being easily broken. Therefore, when hydrogels were soaked with PBS pH 7.4 at 37 °C and stirred at 120 rpm, the cefazolin within the Gel0.50 was released more quickly and seemed similar in Gel0.25. However, the increase in glutaraldehyde added into the gelatin delayed the release of cefazolin. Cefazolin in JGel0.25 was released up to 86.77% after being soaked in PBS with a pH of 7.4 at 37 °C for 8 h. The HPLC chromatogram of cefazolin release in the JGel0.25 for 8 h is shown in Figure S4 (Supplementary Materials Figure S4). The cefazolin release in the JGel0.25 for 8 h was higher than that for BGel0.25 (52.81%) and FGel0.25 (54.04%). Therefore, the diffusion rate is directly proportional to the glutaraldehyde content and depends on the polymer’s molecular weight, the space between the protein chain, and the porous surface area. The release of drugs from hydrogels is also related to the absorption of water molecules into the matrix, followed by the desorption of drug molecules from pores through a diffusion process [49].

## 3. Conclusions

Jellyfish hydrogels were produced through chemical cross-linking using different amounts of glutaraldehyde as a cross-linking agent. The umbrella part of the jellyfish by-products extracted at 60 °C for 24 h (WU24) was used as jellyfish hydrogel, as it presented the highest gel qualities. An increase in the amount of glutaraldehyde added to the hydrogel influenced the gel fraction, the swelling of the hydrogel, and the space between the protein chains in the hydrogel, thereby creating a more robust structure and delaying the release of cefazolin from the hydrogel. The results of the experiment assessing the diffusion mechanism of drug release from the hydrogel, using cefazolin as a model drug, indicated that the rate of drug release from the hydrogels decreased as the ratio of glutaraldehyde increased, demonstrating that the cross-linking reaction between glutaraldehyde and gelatin is an essential factor in regulating the drug release behavior. Jellyfish hydrogels were shown to have controllable drug release, which can be achieved by varying the amount of the chemical cross-linking agent. The cefazolin in JGel0.25—the hydrogel with the largest pore size—was released for 8 h, with a cumulative percentage release of up to 86.77%. Therefore, JGel0.25 is suitable for further application as a drug controlled-release substance. 

## 4. Materials and Methods

**Figure 7:**
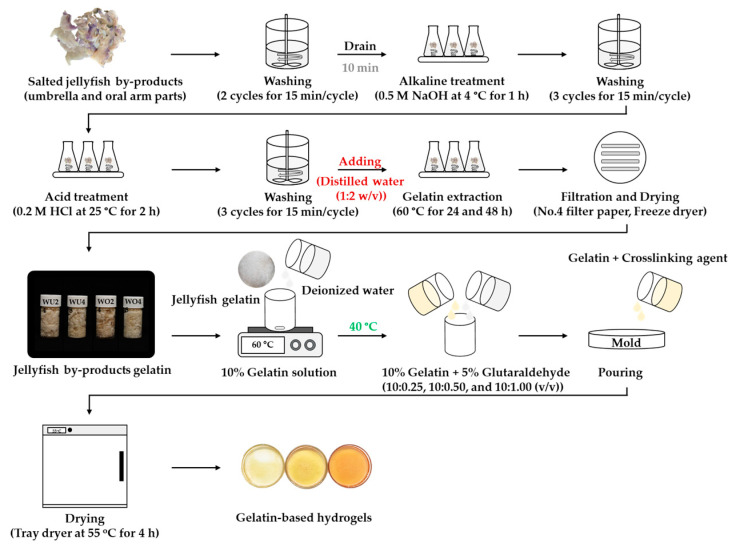
Process diagram of jellyfish hydrogel with glutaraldehyde as a cross-linking agent.

A schematic diagram of the extraction of jellyfish by-product gelatin and the gelatin application as gelatin-based hydrogels is shown in Figure 7.

### 4.1. Preparation of Jellyfish By-Product Gelatin

Salted jellyfish umbrella and oral arm parts (*Lobonema smithii*), as by-products, were received from Mahachai Food and Trading Co., LTD., Samut Sakhon, Thailand. A jellyfish washing machine [50] was used to wash the samples (1000 g) for two cycles at 15 min/cycle. The ratio of sample to tap water was 1:10 (*w*/*v*). After each cycle, the wash water was drained and new water was added. The samples were then drained of water for 10 min [20,50]. After that, the samples were extracted for gelatin.

The jellyfish by-product gelatin was extracted following Lueyot et al. [27] and Charoenchokpanich et al. [51], with slight modifications. Briefly, the desalted jellyfish by-product (100 g) was pre-treated with 0.05 M sodium hydroxide (NaOH; 50% purity) solution (300 mL) at 4 °C for 1 h at 150 rpm using a shaking incubator (WIS-20R, WiseCube, Seoul, Republic of Korea). A machine mechanically washed the alkali-treated jellyfish by-product for three cycles at 15 min/cycle. After alkali pre-treatments, the desalted jellyfish by-product was soaked with 200 mL of 0.2 M hydrochloric acid (HCl; 37% purity) at 150 rpm for 2 h at 25 °C, where the HCl solution was changed every 1 h. Then, the sample was thoroughly washed using a washing machine for three cycles. Next, 200 mL of distilled water was added. Subsequently, the sample was extracted at 60 °C in a water bath shaker (Memmert, Schwabach, Germany) for 24 and 48 h at 80 rpm. The gelatin was then filtered using a Buchner funnel with Whatman No. 4 filter paper [51]. Finally, the gelatin solution was dried using a freeze-dryer (GFD5L, Grisrianthong, Samut Sakhon, Thailand). The jellyfish by-product gelatin samples were then subjected to further analysis.

### 4.2. Preparation of Gelatin-Based Hydrogels

The optimal conditions for jellyfish by-product gelatin extraction were selected and used for the preparation of a gelatin-based hydrogel, based on the obtained gel strength, viscosity, gelling temperature, and melting temperature. First, the commercial gelatin (bovine and fish gelatin) and freeze-dried jellyfish by-product gelatin were dissolved in deionized water at 60 °C for 30 min, until the gelatin had fully solubilized, to obtain a final concentration of 10% (*w*/*v*). The temperature of the gelatin solution was then reduced to 40 °C. After that, 5% glutaraldehyde (25% purity) was subsequently added to the gelatin solution. The mass ratio of gelatin–glutaraldehyde was 10:0.25, 10:0.50, or 10:1.00 (*v*/*v*). The mixed solution was poured into a silicone mold. The hydrogel was left in an oven at 55 °C for 4 h. Then, the hydrogels were washed with 0.1 M glycine for 3 h and deionized water to remove the unreacted glutaraldehyde. Finally, the obtained gelatin-based hydrogel samples were named, including jellyfish hydrogels (JGel0.25, JGel0.50, and JGel1.00), bovine hydrogels (BGel0.25, BGel0.50, and BGel1.00) and fish hydrogels (FGel0.25, FGel0.50, and FGel1.00).

### 4.3. Analysis of Gelatin Properties

#### 4.3.1. Gelatin Yield (%)

The gelatin yield was calculated for all of the extracts on a dry weight basis, using the following Equation (1):

*Gelatin yield* (%) = (*M*_2_/*M*_1_) × 100 (1)

where *M*_1_ is the freeze-dried salted jellyfish by-product weight (g) and *M*_2_ is the freeze-dried jellyfish by-product gelatin weight (g).

#### 4.3.2. Gel Strength

Gelatin gels were prepared at a concentration of 6.67% (*w*/*v*) in deionized water and stirred at 60 °C for 30 min (or until completely dissolved). After that, 6.67% gelatin solution (10 mL) was poured into a vial (5.8 cm in height and 2.7 cm in diameter). The gel sample was kept at 4 °C for 18 h before the analysis. The gel strength of gelatin gel was analyzed using a texture analyzer (TA-XT2i, Stable Micro Systems, Godalming, UK). The measurement was performed as described by Charoenchokpanich et al. [51]. The gel strength of jellyfish by-product gelatin gel was compared to commercial bovine and fish gelatin (McGarrett, JR F&B Co., Ltd., Bangkok, Thailand). 

#### 4.3.3. Viscosity

Viscosity is a property of gel solution that indicates resistance to flow. The viscosity (cP) of the gelatin solution (6.67%) was analyzed using a Brookfield viscometer (DV—II +, BROOKFIELD, Middleboro, MA, USA) at room temperature and 90 rpm.

#### 4.3.4. Thermal Stability

The gelling and melting temperatures of the 6.67% gelatin samples were determined using a stress-controlled rheometer (Kinexus ultra plus, Malvern Instrument, Malvern, UK). A volume of 0.64 mL of gelatin solution was dropped into a stainless-steel concentric cylinder cup (40 mm diameter with a 0.5 mm gap) and covered with paraffin oil. The temperature was swept from 40 °C to 5 °C, holding the temperature at 5 °C for 10 min, then raised to 40 °C at a rate of 2 °C per min, a frequency of 1 Hz, and a shear stress of 1 Pa.

#### 4.3.5. FTIR Spectroscopic Analysis

The functional groups in the gelatin sample were determined using a Perkin-Elmer Spectrum 100 spectrometer (Waltham, MA, USA). The freeze-dried jellyfish by-product and commercial gelatin (bovine and fish gelatin) were mixed with potassium bromide. After that, the sample was pelleted using an MP-1 hydraulic press (JASCO Corporation, Tokyo, Japan). The scanning spectrum ranged from 4000 to 400 cm^−1^, and 4 scans were conducted per sample. Peak deconvolution of the amide I peak (1600–1700 cm^−1^) of the sample was carried out using the Origin 2024 software.

#### 4.3.6. Sodium Dodecyl Sulfate–Polyacrylamide Gel Electrophoresis (SDS-PAGE)

Before performing SDS-PAGE, the gelatin sample was desalted overnight under dialysis. Then, 1000 µg of protein (gelatin) was mixed with a loading buffer (3 M Tris-HCl, pH 6.8; glycerol; 10% *w*/*v* SDS; 1% *w*/*v* bromophenol blue; and 5% 2-mercaptoethanol, DTT) at a ratio of 1:2 (*v*/*v*). The mixed solution was heated in a water bath at 95 °C for 3 min before loading onto 5% stacking gel and 12% separating gel. Each cooled sample was loaded onto the gel and subjected to an electrophoresis unit (Mini-slab size electrophoresis system, ATTO, Tokyo, Japan) with a constant current of 50 V in vertical slab gels. The molecular weight of the gelatin protein was estimated using a high molecular weight protein marker (11–250 kDa) (PageRulerTM Plus Prestained protein ladder, Thermo Fisher Scientific, Rockford, IL, USA) [52].

#### 4.3.7. Protein Identification of Gelatin Using LC-MS/MS

The commercial gelatins and jellyfish by-product gelatin were treated according to the method of Lueyot et al. [27] and loaded onto LC-MS/MS for analysis. Briefly, the gelatin sample was digested with mass spectrometer-grade trypsin at a ratio of 1:20 for 24 h at 37 °C, and was dried using vacuum centrifugation (SpeedVac concentrator, Eppendorf, Hamburg, Germany). After that, the sample was dissolved with 0.1% formic acid (98% purity) for LC-MS/MS analysis.

The peptide samples were injected into an Ultimate 3000 Nano/Capillary LC System (Thermo Scientific, Germering, Germany) coupled to a Hybrid quadrupole Q-TOF impact II™ (Bruker Daltonics, Bremen, Germany). They were loaded onto a C18 reversed-phase column packed (15 cm length × 75 µm diameter) with an Acclaim PepMap^®^ RSLC C18, 3 μm, 100 Å, nanoViper (Thermo Scientific, Bremen, Germany). The C18 column was enclosed in a thermostat oven set to 40 °C. The tryptic peptides were separated using a linear gradient of 5–55% solvent B (0.1% formic acid in 80% acetonitrile) at a flow rate of 300 μL/min over 45 min, following equilibration with solvent A (0.1% formic acid in water). Electrospray ionization was carried out at 1.6 kV using CaptiveSpray for mass identification. Nitrogen was used as a drying gas at a flow rate of approximately 50 L/h, which was used as the collision gas to produce collision-induced dissociation (CID) product ion mass spectra. In the positive-ion mode, mass spectra (MS) and MS/MS spectra were acquired at 2 Hz over the range (*m*/*z*) 150–2200 (Compass 1.9 software, Bruker Daltonics).

Individual samples were bioinformatically quantified using the MaxQuant v2.4.10.0 software, and their MS/MS spectra were matched to the UniProt database using the Andromeda search engine [53]. The protein data set comprised alpha-1 and alpha-2 chains, bovine collagen, bovine gelatin, fish collagen, and fish gelatin, retrieved from the UniProt database. All possible contaminants that did not match any UPS1 protein were removed from the data set using Perseus version 2.0.6.0 [54]. Mass intensities were log_2_-transformed. Perseus imputed missing values with a constant value (zero) [55]. The visualization and statistical analyses of the LC-MS data, including heatmap and principal component analysis (PCA), were conducted using MetaboAnalyst 6.0.

### 4.4. Analysis of Gelatin-Based Hydrogel Properties

#### 4.4.1. Gel Fraction

The hydrogel (0.2 g; *W_i_*) was soaked in distilled water (100 mL) and stirred at 50 rpm for 48 h at 30 °C. The hydrogel sample was then vacuum-filtered and dried at 60 °C for 24 h. Then, the sample was weighted (*W_d_*). The gel fraction percentages were calculated using Equation (2):

*Gel fraction* (%) = (*W_d_*/*W_i_*) × 100 (2)

where *W_d_* is the weight of the insoluble dry hydrogel (g) and *W_i_* is the initial weight of a dry hydrogel (g). 

#### 4.4.2. Gel Porosity

A quantity of 0.2 g of hydrogel (*W*_1_) was immersed in 100 mL of ethanol at 25 °C for 24 h. Then, the hydrogel sample was filtered and weighed (*W*_2_). The gel porosity and volume of porosity were calculated using the following Equations (3) and (4), respectively:

*Gel porosity* (%) = [(*W*_2_ − *W*_1_)/(*ρ_ethanol_* × *V_G_*)] × 100 (3)

*Volume of porosity* (cm^3^) *=* (*W*_2_ − *W*_1_)/*ρ_ethanol_* (4)

where *W*_1_ is the weight of the hydrogel (g), *W*_2_ is the weight of hydrogel after soaking and filtration (g), *ρ_ethanol_* is the density of ethanol (0.789 g/cm^3^), and *V_G_* is the hydrogel volume (cm^3^).

#### 4.4.3. Microstructure

Each hydrogel was cut into 5 × 5 mm samples. Then, the samples were dehydrated with a serial concentration of ethanol (30% to 100%) for 15 min. After that, the samples were 100% gold sputter-coated (Sputterm coater SPI-Module, West Chester, PA, USA). Scanning electron microscopy (SEM) was conducted to visualize the microstructure of hydrogels.

#### 4.4.4. Gel swelling

The hydrogel sample (0.2 g) was soaked in phosphate-buffered saline (PBS) solutions pH 7.4 (100 mL) at 30 °C for 24 h and stirred at 50 rpm. The hydrogel weight was recorded after swelling at 1, 2, 4, 6, 8, 24, and 48 h. The swelling percentage of the hydrogel can be calculated using the following Equation (5):

*Gel swelling* (%) = [(*W_t_* − *W_o_*)/*W_o_*] × 100 (5)

where *W_t_* is the weight of swelling hydrogel at a different certain time and *W_o_* is the initial weight of the dry hydrogel.

#### 4.4.5. In Vitro Drug Release

Hydrogels were prepared using in situ drug loading methods, through adding 1 mL cefazolin (97.12% purity) at 100 µg/mL during hydrogel gelation. The drug-loaded hydrogel composites were immersed in PBS with a pH of 7.4 (100 mL) at 37 °C and stirred at 120 rpm. The drug release was tested at 1, 2, 3, 4, 5, 6, 7, 8, 24, and 48 h. At predetermined time intervals, aliquots (1.5 mL) of the release medium were removed, and an identical volume of fresh medium was added. The drug concentration in the release medium was quantified using High-Performance Liquid Chromatography (HPLC), comprising a column C18 (Agilent, 5 μm, 250 × 4.6 mm), mobile phase (methanol: 0.1% acetic acid at a ratio of 20:80), and flow rate of 0.6 mL/min at 25 °C. The chromatograms were detected at 245 nm. Cefazolin was quantified by calibrating the cefazolin standard curve. Cumulative percentage release (*CPR*) was calculated using the following Equation (6):

*CPR* (%) = (*W_ct_*/*W_c_*) × 100 (6)

where *W_ct_* is the amount of cefazolin released from the hydrogel at the time, and *W_c_* is the amount of cefazolin loaded onto the hydrogel. 

### 4.5. Statistical Analysis

Statistical Package for Social Science (SPSS) 22.0 was used to perform the one-way analysis of variance (ANOVA), and a comparison of means (*p* < 0.05) was carried out using Duncan’s multiple range test.

## Figures and Tables

**Table 1:** Physical properties of commercial bovine gelatin, commercial fish gelatin, and jellyfish by-product gelatin with different parts of the jellyfish and extraction times.

Gelatin Sample	Gel Strength * (g)	Viscosity * (cP)	Gelling Temperature * (°C)	Melting Temperature * (°C)
BG	846.31 ± 0.70 ^a^	30.06 ± 0.92 ^a^	22.22 ± 0.47 ^a^	30.23 ± 0.08 ^a^
FG	625.55 ± 0.40 ^b^	25.54 ± 0.02 ^b^	18.69 ± 0.05 ^b^	25.23 ± 0.02 ^b^
WU24	460.02 ± 0.21 ^c^	24.45 ± 0.35 ^c^	12.70 ± 0.67 ^c^	22.48 ± 0.12 ^c^
WO24	449.11 ± 0.86 ^d^	23.62 ± 0.55 ^d^	11.66 ± 0.85 ^c^	22.34 ± 1.02 ^c^
WU48	412.63 ± 0.39 ^e^	22.44 ± 0.19 ^e^	9.93 ± 0.88 ^d^	20.11 ± 0.90 ^d^
WO48	408.08 ± 0.52 ^f^	20.61 ± 0.33 ^f^	8.56 ± 0.20 ^e^	18.18 ± 0.64 ^e^

* Different superscript letters in the same column indicate a significant difference (*p* < 0.05).

**Table 2:** Gel fraction percentage and gel porosity of gelatin-based hydrogels.

Sample		Gel Fraction * (%)	Gel Porosity * (%)
Bovine hydrogels (BGel)	BGel0.25	14.97 ± 0.46 ^c^	1.92 ± 0.09 ^b^
	BGel0.50	15.08 ± 0.82 ^c^	1.88 ± 0.05 ^bc^
	BGel1.00	19.14 ± 0.60 ^a^	1.73 ± 0.01 ^d^
Fish hydrogels (FGel)	FGel0.25	13.02 ± 0.16 ^d^	2.15 ± 0.08 ^a^
	FGel0.50	16.27 ± 0.25 ^b^	1.90 ± 0.02 ^b^
	FGel1.00	17.32 ± 0.59 ^b^	1.79 ± 0.04 ^cd^
Jellyfish hydrogels (JGel)	JGel0.25	12.58 ± 0.49 ^d^	2.20 ± 0.08 ^a^
	JGel0.50	14.30 ± 0.55 ^c^	1.91 ± 0.04 ^b^
	JGel1.00	17.20 ± 0.97 ^b^	1.83 ± 0.05 ^bcd^

* Different superscript letters in the same column indicate a significant difference (*p* < 0.05).

## Data Availability

All data presented in this study are available on request from the corresponding author.

## Supplementary Materials

The following supporting information can be downloaded at https://www.mdpi.com/article/10.3390/gels10040271/s1, Table S1: Peak wavenumbers of commercial gelatin and jellyfish by-product gelatin with different parts of jellyfish and extraction times; Table S2: Comparison of secondary structure from the band of amide I (1600–1700 cm^−1^); Table S3: Protein names and sequence of commercial gelatins (bovine, BG; and fish, FG) and jellyfish by-product gelatin with different parts of jellyfish and extraction times; Table S4: Gel swelling of bovine hydrogels; Table S5: Gel swelling of fish hydrogels; Table S6: Gel swelling of jellyfish hydrogels; Table S7: Cumulative percentage release of bovine hydrogels; Table S8: Cumulative percentage release of fish hydrogels; Table S9: Cumulative percentage release of jellyfish hydrogels; Figure S1: Dynamic viscoelastic profile changes in commercial gelatins (bovine, BG; and fish, FG) and jellyfish by-product gelatins during gelling and melting: (A) during gelling and (B) during melting; Figure S2: Schiff base linkages are created during a reaction between the amino group of lysine and the carbonyl groups of glutaraldehyde; Figure S3: Adsorption of cefazolin solution in hydrogels; Figure S4: HPLC chromatogram of cefazolin at 100 ppm (A) and release of cefazolin in the jellyfish hydrogel (JGel0.25; 10: 0.25 (*v*/*v*)) for 8 h (B).

## Abbreviations

**BG**	Commercial bovine gelatin
**BGel**	Bovine hydrogels
**BGel0.25**	Bovine hydrogels with a ratio of commercial bovine gelatin: glutaraldehyde at 10:0.25 (*v*/*v*)
**BGel0.50**	Bovine hydrogels with a ratio of commercial bovine gelatin: glutaraldehyde at 10:0.50 (*v*/*v*)
**BGel1.00**	Bovine hydrogels with a ratio of commercial bovine gelatin: glutaraldehyde at 10:1.00 (*v*/*v*)
**FG**	Commercial fish gelatin
**FGel**	Fish hydrogels
**FGel0.25**	Bovine hydrogels with a ratio of commercial fish gelatin: glutaraldehyde at 10:0.25 (*v*/*v*)
**FGel0.50**	Bovine hydrogels with a ratio of commercial fish gelatin: glutaraldehyde at 10:0.50 (*v*/*v*)
**FGel1.00**	Bovine hydrogels with a ratio of commercial fish gelatin: glutaraldehyde at 10:1.00 (*v*/*v*)
**JGel**	Jellyfish hydrogels
**JGel0.25**	Jellyfish hydrogels with a ratio of gelatin extracted from the umbrella part of desalted jellyfish by-product for 24 h: glutaraldehyde at 10:0.25 (*v*/*v*)
**JGel0.50**	Jellyfish hydrogels with a ratio of gelatin extracted from the umbrella part of desalted jellyfish by-product for 24 h: glutaraldehyde at 10:0.50 (*v*/*v*)
**JGel1.00**	Jellyfish hydrogels with a ratio of gelatin extracted from the umbrella part of desalted jellyfish by-product for 24 h: glutaraldehyde at 10:1.00 (*v*/*v*)
**WO24**	Gelatin extracted from the oral arm part of desalted jellyfish by-product for 24 h
**WO48**	Gelatin extracted from the oral arm part of desalted jellyfish by-product for 48 h
**WU24**	Gelatin extracted from the umbrella part of desalted jellyfish by-product for 24 h
**WU48**	Gelatin extracted from the umbrella part of desalted jellyfish by-product for 48 h
