# Supplementary material for: Exploring the Model of Cefazolin Released from Jellyfish Gelatin-Based Hydrogels as Affected by Glutaraldehyde
Source: Gels. 2024 Apr 17;10(4):271. doi: 10.3390/gels10040271 (PMC11048929; doi:10.3390/gels10040271)
Supplement: Supplementary file 1 [file gels-10-00271-s001.zip › gels-2954044-supplementary.pdf]

Table S1. Peak wavenumbers of commercial gelatin and jellyfish by-product gelatin with different parts of jellyfish and extraction times.

Region	Peak wavenumber (cm^{-1})					
	BG	FG	WU24	WO24	WU48	WO48
Amide A	3438.65	3424.21	3353.99	3412.81	3422.64	3403.23
Amide B	2918.86	2927.48	2930.04	2923.03	2928.30	2928.45
Amide I	1607.43	1619.56	1626.89	1628.54	1630.84	1630.72
Amide II	1561.36	1567.14	1558.84	1559.08	1555.08	1550.02
Amide III	1240.08	1239.84	1239.29	1238.63	1229.95	1229.07

Table S2. Comparison of secondary structure from the band of amide I (1600-1700 cm^{-1}).

**Sample**	β-sheet (%)	Random coil (%)	α-helix (%)	β-turn (%)
BG	30.26	22.95	0.47	46.33
FG	10.99	6.09	9.06	73.87
WU24	4.24	11.81	31.62	52.34
WO24	5.86	58.41	3.75	31.98
WU48	15.85	26.60	24.90	32.65
WO48	29.89	49.48	16.77	3.85

Table S3. Protein names and sequence of commercial gelatins (bovine, BG; and fish, FG) and jellyfish by-product gelatin with different parts of jellyfish and extraction times.

Protein names	Peptide sequences	BG	FG	WU24	WO24	WU48	WO48
Collagen alpha-1(XXI) chain	PGDSGEPGDKG	+	+	+	+	+	+
Collagen alpha-1(XXIV) chain	DCQREISD	+	+		+		
Collagen alpha-1(XXVII) chain B	GGKGGAGR	+	+	+	+		+
Collagen alpha-1(II) chain	AKGSAGAP	+	+	+	+	+	+
Collagen alpha-1(XI) chain A	DGEPGEKGEDGESGQ	+	+	+	+		
Collagen alpha-1(XXII) chain	QAGAQGEP	+	+	+			
Collagen alpha-1(XXVIII) chain B	GEPGLTVSCGECIS	+	+	+	+		
Collagen alpha-1(IX) chain A	GEAGPSGG	+	+	+	+		
Collagen alpha-1(XII) chain	HVFEVEDF	+	+		+		
Collagen alpha-1(I) chain B	AAGATGFP	+	+	+	+		
Collagen alpha-1(VII) chain	ACGGSGPD	+	+	+	+	+	+
Collagen alpha-1(IV) chain	GMPGKDGEPG	+	+	+		+	
Collagen alpha-1(I) chain A	DGDVGAPGASGP	+		+			
Collagen alpha-1(XXVIII) chain A	GPAGPSGMQGFP	+	+	+	+	+	+
Collagen alpha-1(XI) chain	EEDSYYYEYPYYDDADAK PLEPTT	+	+	+	+		
Collagen alpha-1(XVI) chain	LYVTYSHGADAGEQ	+	+	+	+	+	+
Collagen alpha-1(I) chain	AGAAGPAQGDP	+		+			
Collagen alpha-1(XVIII) chain A	PGHPGHSS	+	+				
Collagen alpha-2(I) chain	GDPGPPGL	+	+	+	+	+	+
Collagen alpha-2(V) chain-like	GFPGSPGG	+					+
Collagen alpha-2(VIII) chain	LPCGGWNCECAF	+					
Collagen alpha-2(IX) chain	VGKPGGKG	+	+	+	+	+	+
Collagen alpha-2(XI) chain	GPPGPPGKRGPPGT	+	+	+	+	+	
Collagen alpha-3(VI) chain	GARGYNGE	+	+				
Collagen alpha-4(IV) chain-like	GPPGPPGTCGEH	+	+	+		+	
Collagen alpha-5(IV) chain	GLPGFPGTP	+	+	+		+	+
Collagen alpha-6(VI) chain	KFVKAFISSV	+		+			
Collagen alpha-6(IV) chain	GFCACDGG	+	+	+			

"+" stands for the existence of peptide.

Table S4. Gel swelling of bovine hydrogels.

Sample	Gel swelling* (%)							
	0 h	1 h	2 h	4 h	6 h	8 h	24 h	48 h
BGel0.25**	1.02 ± 0.02^{Hb}	22.24 ± 0.76^{Ga}	45.76 ± 0.65^{Fa}	57.30 ± 0.56^{Ea}	76.83 ± 0.38^{Da}	95.60 ± 0.57^{Ca}	150.03 ± 0.68^{Ba}	166.53 ± 0.45^{Aa}
BGel0.50**	1.08 ± 0.02^{Ha}	11.48 ± 0.50^{Gb}	19.38 ± 0.73^{Fb}	21.98 ± 0.93^{Eb}	34.05 ± 0.90^{Db}	47.27 ± 0.64^{Cb}	75.28 ± 0.76^{Bb}	82.33 ± 0.57^{Ab}
BGel1.00**	1.07 ± 0.01^{Ha}	2.36 ± 0.30^{Gc}	4.59 ± 0.57^{Fc}	6.68 ± 0.24^{Ec}	9.27 ± 0.17^{Dc}	11.95 ± 0.22^{Cc}	25.91 ± 0.52^{Bc}	29.71 ± 0.64^{Ac}

*Different superscripts (A-H) in the same column indicate a significant difference ($p<0.05$).
**Different superscripts (a-c) in the same row indicate a significant difference ($p<0.05$).

Table S5. Gel swelling of fish hydrogels.

Sample	Gel swelling* (%)							
	0 h	1 h	2 h	4 h	6 h	8 h	24 h	48 h
FGel0.25**	1.07 ± 0.02^{Ha}	30.04 ± 1.72^{Ga}	50.94 ± 0.75^{Fa}	71.43 ± 0.48^{Ea}	87.49 ± 0.94^{Da}	100.05 ± 0.42^{Ca}	155.00 ± 0.65^{Ba}	171.15 ± 0.59^{Aa}
FGel0.50**	1.05 ± 0.01^{Hb}	12.04 ± 0.84^{Gb}	23.17 ± 0.92^{Fb}	25.67 ± 0.43^{Eb}	43.56 ± 0.46^{Db}	59.53 ± 0.65^{Cb}	83.32 ± 0.55^{Bb}	89.90 ± 0.58^{Ab}
FGel1.00**	1.07 ± 0.01^{Ha}	3.78 ± 0.63^{Gc}	5.91 ± 0.40^{Fc}	7.83 ± 0.89^{Ec}	10.27 ± 0.40^{Dc}	14.07 ± 0.48^{Cc}	31.13 ± 0.29^{Bc}	34.64 ± 0.64^{Ac}

*Different superscripts (A-H) in the same column indicate a significant difference ($p<0.05$).
**Different superscripts (a-c) in the same row indicate a significant difference ($p<0.05$).

Table S6. Gel swelling of jellyfish hydrogels.

Sample	Gel swelling* (%)							
	0 h	1 h	2 h	4 h	6 h	8 h	24 h	48 h
JGel0.25**	1.03±0.01^{Hb}	34.10±0.77^{Ga}	54.43±0.65^{Fa}	72.90±0.53^{Ea}	94.50±0.45^{Da}	104.05±0.72^{Ca}	165.74±0.31^{Ba}	174.06±0.63^{Aa}
JGel0.50**	1.03±0.01^{Hb}	12.80±0.48^{Gb}	26.38±0.58^{Fb}	29.78±0.38^{Eb}	45.99±0.24^{Db}	65.08±0.74^{Cb}	93.49±0.69^{Bb}	95.30±0.57^{Ab}
JGel1.00**	1.09±0.01^{Ha}	3.82±0.71^{Gc}	6.74±0.80^{Fc}	8.28±0.29^{Ec}	11.25±0.50^{Dc}	18.04±0.66^{Cc}	36.00±0.11^{Bc}	38.47±0.10^{Ac}

*Different superscripts (A-H) in the same column indicate a significant difference ($p<0.05$).
**Different superscripts (a-c) in the same row indicate a significant difference ($p<0.05$).

Table S7. Cumulative percentage release of bovine hydrogels.

Time (h)	The cumulative percentage release* (%)		
	BGel0.25	BGel0.50	BGel1.00
1**	25.98±0.65^{Ja}	25.61±0.24^{Ia}	13.10±0.86^{Ib}
2**	31.75±0.43^{Ia}	27.83±0.33^{Hb}	18.75±0.17^{Hc}
3**	37.32±0.53^{Ha}	31.68±1.26^{Gb}	20.40±0.58^{Gc}
4**	39.69±0.09^{Ga}	37.30±1.35^{Fb}	24.53±0.80^{Fc}
5**	44.01±0.57^{Fa}	38.72±0.32^{Fb}	28.59±0.53^{Ec}
6**	47.19±0.69^{Ea}	41.23±0.29^{Eb}	30.57±0.26^{Dc}
7**	49.53±0.99^{Da}	44.05±0.60^{Db}	34.18±0.45^{Cc}
8**	52.82±0.15^{Ca}	49.45±1.26^{Cb}	35.65±0.23^{Bc}
24**	58.93±0.75^{Ba}	56.30±1.78^{Bb}	36.52±0.50^{Bc}
48**	62.25±2.23^{Aa}	60.64±0.22^{Aa}	39.94±0.65^{Ab}

*Different superscripts (A-J) in the same column indicate a significant difference ($p<0.05$).
**Different superscripts (a-c) in the same row indicate a significant difference ($p<0.05$).

Table S8. Cumulative percentage release of fish hydrogels.

Time (h)	The cumulative percentage release* (%)		
	FGel0.25	FGel0.50	FGel1.00
1**	33.34±2.04^{Ha}	22.75±0.98^{Ib}	20.12±0.16^{Jc}
2**	38.73±1.65^{Ga}	31.10±0.43^{Hb}	25.49±1.61^{Ic}
3**	40.30±0.14^{Ga}	32.60±0.94^{Hb}	29.05±0.58^{Hc}
4**	44.25±0.55^{Fa}	39.87±1.58^{Gb}	31.42±0.30^{Gc}
5**	46.15±0.93^{Ea}	46.15±1.08^{Fa}	33.960.61^{Fb}
6**	48.09±0.32^{Da}	48.48±0.63^{Ea}	35.70±0.52^{Eb}
7**	49.78±0.46^{Da}	50.73±0.91^{Da}	38.66±0.68^{Db}
8**	54.05±0.38^{Ca}	52.73±0.84^{Cb}	41.30±0.08^{Cc}
24**	58.41±0.98^{Ba}	57.71±0.27^{Ba}	44.84±0.51^{Bb}
48**	67.24±0.94^{Aa}	64.55±0.38^{Ab}	47.52±0.86^{Ac}

*Different superscripts (A-J) in the same column indicate a significant difference ($p<0.05$).
**Different superscripts (a-c) in the same row indicate a significant difference ($p<0.05$).

Table S9. Cumulative percentage release of jellyfish hydrogels.

Time (h)	The cumulative percentage release* (%)		
	JGel0.25	JGel0.50	JGel1.00
1**	27.89±1.30^{Ia}	24.37±2.24^{Ia}	18.28±4.31^{Gb}
2**	55.62±0.82^{Ha}	48.86±2.12^{Hb}	34.01±2.83^{Fc}
3**	65.32±3.15^{Ga}	57.71±1.11^{Gb}	37.95±0.12^{Ec}
4**	70.33±1.58^{Fa}	64.32±1.82^{Fb}	38.47±0.33^{DEc}
5**	72.90±1.00^{Ea}	68.05±0.44^{Eb}	40.37±0.62^{CDEc}
6**	76.71±1.62^{Da}	70.71±1.90^{Db}	41.28±0.35^{CDc}
7**	79.35±0.24^{Ca}	75.00±1.00^{Cb}	42.23±0.82^{BCc}
8**	86.77±0.89^{Ba}	80.26±0.62^{Bb}	43.44±0.45^{BCc}
24**	88.82±0.34^{Ba}	85.65±0.90^{Ab}	45.15±0.79^{Bc}
48**	91.50±0.33^{Aa}	87.09±0.83^{Ab}	50.19±1.25^{Ac}

*Different superscripts (A-I) in the same column indicate a significant difference ($p<0.05$).
**Different superscripts (a-c) in the same row indicate a significant difference ($p<0.05$).

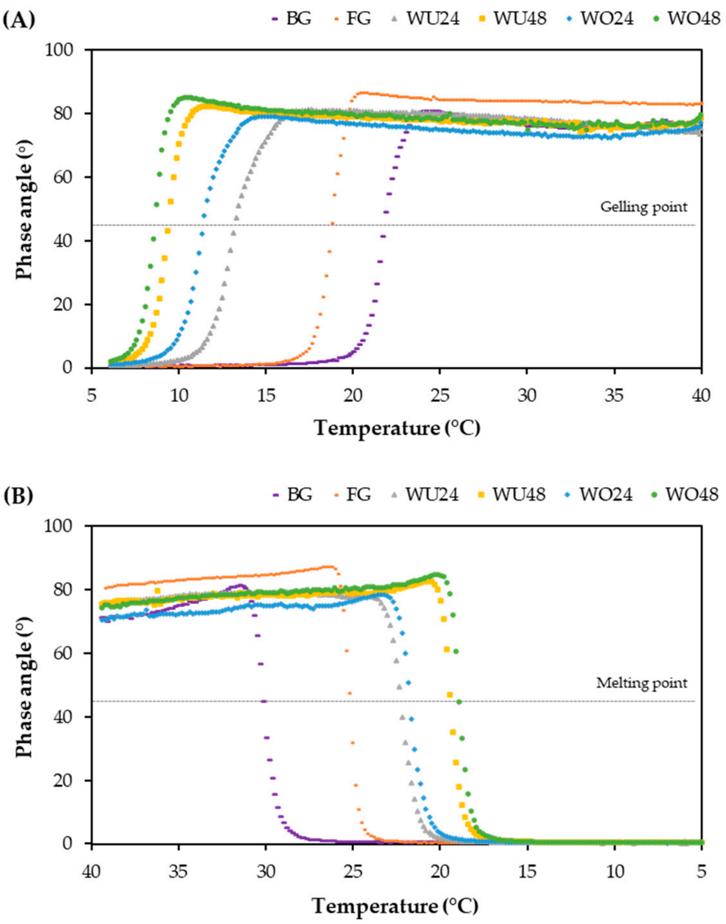

Figure S1. Dynamic viscoelastic profile changes in commercial gelatins (bovine, BG; and fish, FG) and jellyfish by-product gelatins during gelling and melting (A) during gelling and (B) during melting.

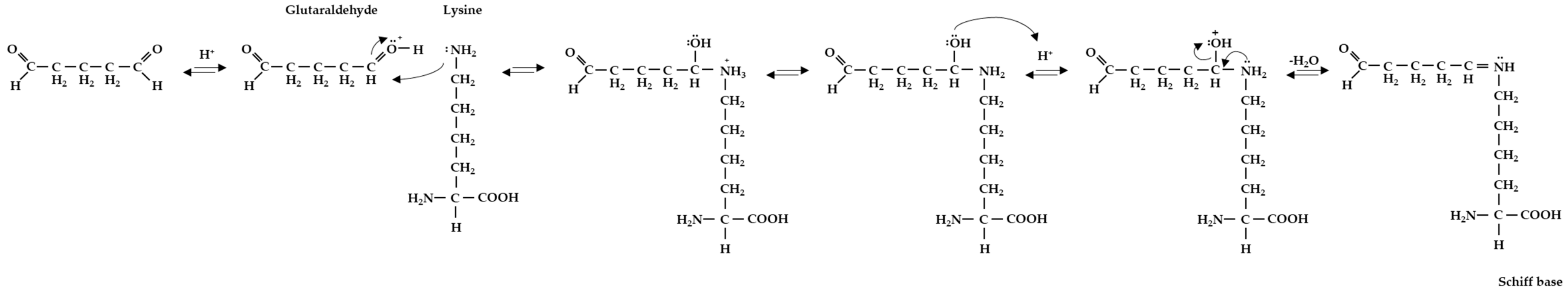

Figure S2. Schiff base linkages are created by a reaction between the amino group of lysine and the carbonyl groups of glutaraldehyde.

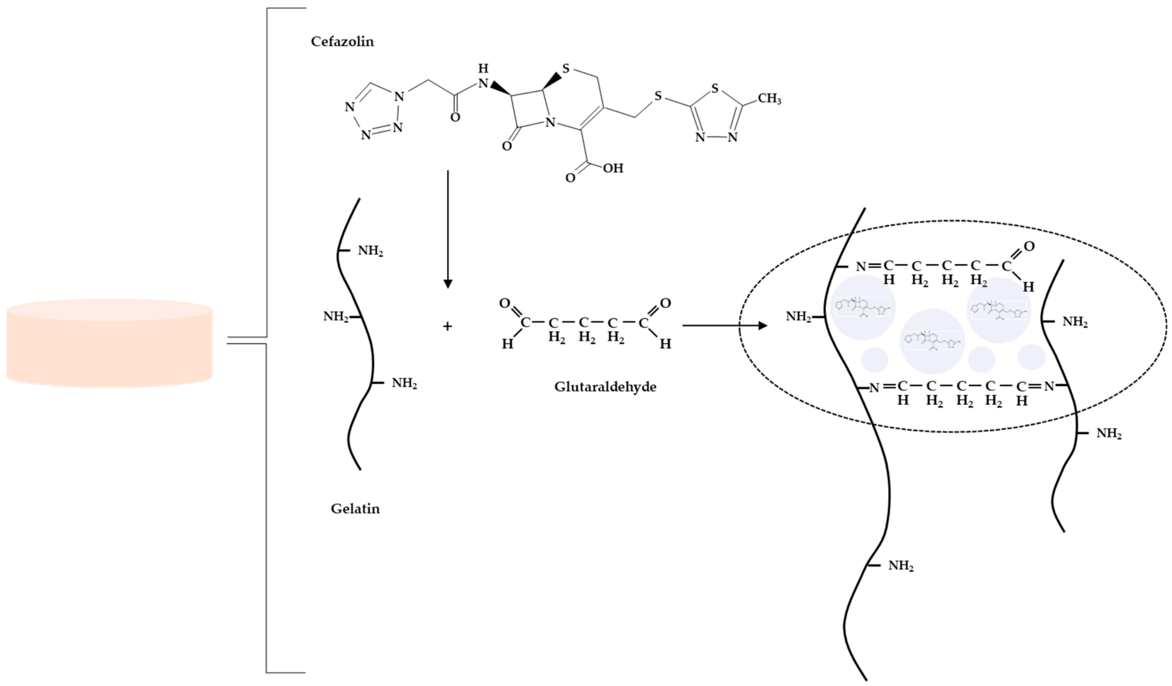

Figure S3. Adsorption of cefazolin solution in hydrogels.

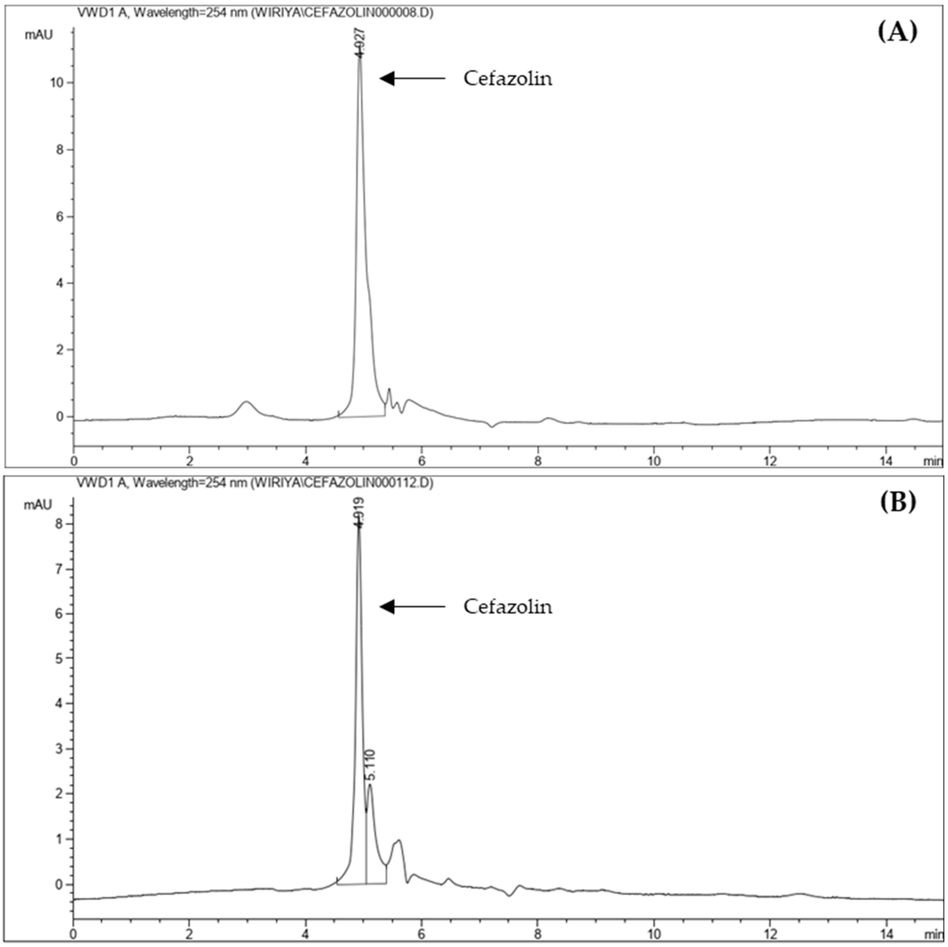

Figure S4. HPLC chromatogram of cefazolin at 100 ppm (A) and release of cefazolin in the jellyfish hydrogel (JGel0.25; 10: 0.25 (v/v)) for 8 h (B).
